# Shared Resource Management in the Cancer Center Environment: Beyond Operational Excellence

**DOI:** 10.7171/001c.169030

**Published:** 2026-09-10

**Authors:** Agnes Viale, Marisa Dunigan, Patrick Badua, Nyree Cabrera-Lugo, Kety Huberman

**Affiliations:** 1 Shared Resource Management Memorial Sloan Kettering Cancer Center

**Keywords:** Shared Resource Management, NCI designated Cancer Centers, Shared Resources, Cores, CCSG, NIH funding, cancer center resilience, translational research

## Abstract

Shared Resource Management (SRM) is critical to demonstrating the scientific, operational, and strategic value of shared resources (SRs) within NCI-designated Cancer Centers, particularly during quinquennial Cancer Center Support Grant (CCSG) renewal. SRs provide advanced technologies, specialized expertise, and coordinated infrastructure that support basic, translational, and clinical research. Within this framework, SRM advances three core aims: sustaining state-of-the-art resources, centralizing operational and financial management, and fostering innovation to meet emerging scientific needs. This paper examines SRM through the following aims, highlighting its importance for CCSG preparation, institutional competitiveness, and adaptation to a changing NIH funding landscape. Despite evolving review expectations and funding uncertainty, effective SRM remains essential to cancer center resilience and research excellence.

## Introduction

**Figure:**
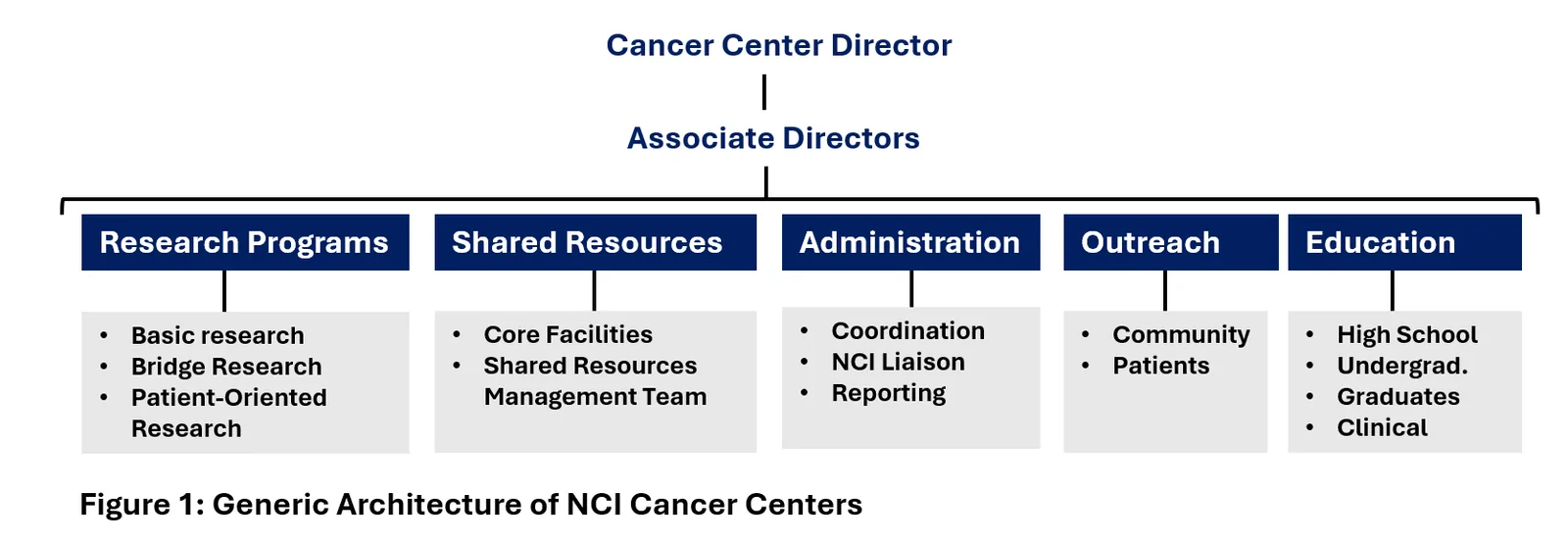


The ecosystems of National Cancer Institute (NCI)-designated cancer centers (Figure 1) operate on a quinquennial cycle. In the year preceding a competitive renewal, all hands are on deck, particularly the shared resources management (SRM) team, whose mission is to assemble a compelling document demonstrating how shared resources (SRs) are central to the scientific, operational, and strategic success of modern cancer centers. While some evaluation metrics for SRs are consistent across institutions, SRs within NCI-designated cancer centers are characterized by additional layers of complexity.

Core facilities provide investigators with access to advanced technologies, specialized expertise, and coordinated infrastructures to support basic, translational, and clinical research. Within the framework of each Cancer Center Support Grant (CCSG), which is administered by the NCI, SRs are rigorously evaluated as essential components of institutional research capacity.

For an NCI-designated cancer center, SRM teams are structured to support scientific excellence, operational efficiency, and long-term sustainability of the core facilities. SRM teams are guided by three overarching aims: (1) building and maintaining state-of-the-art SRs; (2) facilitating centralized management of operations, finances, and personnel; and (3) fostering innovation to meet future scientific challenges. This paper examines SRM teams through the lens of these three aims, emphasizing their role in CCSG preparation and institutional competitiveness.

Since January 2025, the evolving NIH funding landscape (including shifts in priorities, support mechanisms, and guidance) has introduced additional uncertainty, with the Notice of Funding Opportunity (NOFO) and review format subject to ongoing change. SRM teams are partially responsible for integrating these elements into a cohesive narrative that aligns with the criteria defined in the latest NOFO.^1^ Although the ecosystem may feel somewhat fragile at the moment, it is important to remember that it has successfully navigated many shifts in its landscape over the past 50 years. As long as we remain focused on our core mission, this ecosystem will emerge stronger and well positioned for the future of cancer research.

## Shared Resources in the Context of CCSGs

CCSGs support the infrastructure of NCI-designated cancer centers, including leadership, research programs, and SRs. Unlike project-based funding, CCSGs emphasize organizational excellence and the ability to support collaborative and multidisciplinary research.^1^ SRs are therefore evaluated based on their scientific impact, utilization, quality, and sustainability.

While the number of SRs varies across cancer centers, they generally represent a large-scale enterprise, encompassing up to 20 core facilities, operating budgets in the tens of millions of dollars, and hundreds of core scientists. These resources support a wide spectrum of research activities and are deeply integrated into each cancer center’s scientific mission.

## Building and Maintaining State-of-the-Art Shared Resources

The first goal of SRM teams in NCI-designated cancer centers is to build and maintain state-of-the-art SRs. Centralized management teams focus on ensuring that SRs are cutting edge and aligned with the evolving needs of cancer center members. This includes both maintaining existing capabilities and developing new resources across the cancer research spectrum. At a minimum, SRs must encompass a diverse array of technologies, including genomics, bioinformatics, imaging, biostatistics, and biobanking. These facilities provide services that are often too complex or too resource intensive for individual laboratories to sustain independently. By centralizing these capabilities, cancer centers can ensure access to high-quality, standardized methodologies that enhance reproducibility and efficiency.

Once established, core facilities are constantly assessed for their relevance to the cancer center. SRM teams are responsible for developing tools for this evaluation. Through surveys, trend analyses, and direct engagement with investigators, the scientific relevance of a core facility is determined, thus enabling rapid adaptation to emerging research priorities. For example, the integration of new cores and the consolidation or discontinuation (i.e., “sunsetting”) of underutilized services reflect a dynamic approach to resource management.

Over time, the impact of SRs must be tangible. In a five-year grant cycle, SRs typically contribute to thousands of publications. In particular, many of these publications are in high-impact journals, thus demonstrating the critical role of SRs in advancing scientific discovery.

Other key indicators include utilization for both internal and external users, academic collaborators, industry partners, as well as the total number of services provided. Clinical trial support highlights the role of SRs in advancing translational and clinical research, which is particularly important for comprehensive cancer centers. Financial performance is assessed through grant funding and overall operational budget management. In the context of CCSGs, such metrics are essential indicators of scientific value and institutional strength.

**Figure:**
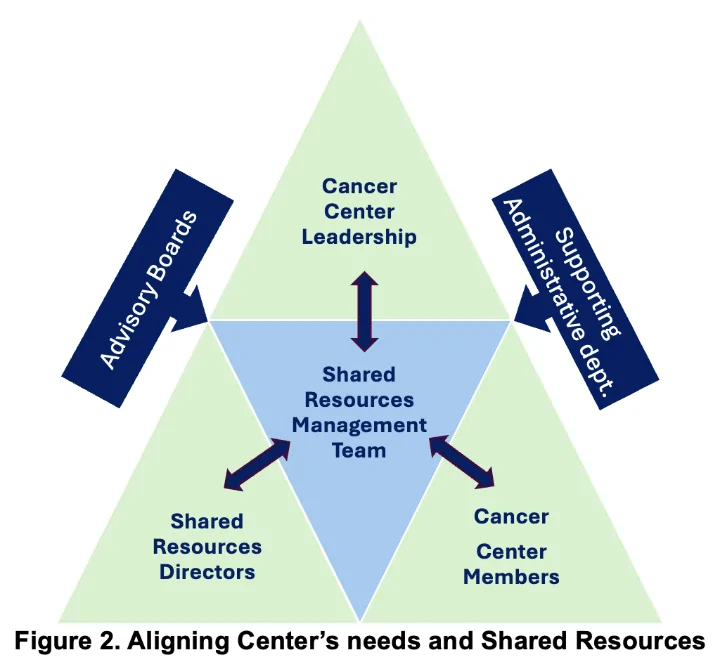


The SRM team is responsible for the aligning the needs of cancer center members with all SRs. The team must continuously engage with multiple stakeholders, including the cancer center director, associate directors, and the administration (Figure 2).

## Centralized Management of Operations, Finances, and Personnel

The second goal of SRM teams is to ensure the efficiency, accountability, and sustainability of their operation. In the past 20 years, most NCI-designated cancer centers have moved away from private or departmental SRs and adopted a centralized oversight of all SRs, which entails standardized policies and coordinated operational activities across core facilities. This strategy increased compliance and decreased conflicts of interest.

Centralized systems streamline access to services, scheduling, and project initiation. Commercial or homebrewed platforms facilitate user interaction with SRs, while standardized policies such as pricing, access, and conflict resolution enhance compliance with CCSG guidelines.

Financial sustainability is a critical requirement for CCSG evaluation. Cancer centers typically employ a combination of cost recovery, institutional support, and financial monitoring tools to maintain balanced operations. Innovative platforms such as real-time financial dashboards and invoice tracking systems provide transparency and enable data-driven decision-making.

Cost-effectiveness strategies include bulk purchasing, service consolidation, and the implementation of lean methodologies to reduce waste and improve efficiency. There are many examples of process optimization initiatives that have led to measurable cost savings and improved operational performance.^2^

The management of highly skilled personnel is another cornerstone of SRM teams. Since a seminal publication by Chitty et al. in 2023,^3^ institutions have implemented structured career development pathways, succession planning, and professional training programs to attract and retain talent. These efforts ensure continuity of expertise and support long-term institutional stability.

This centralized approach through the SRM team is not exclusive to NCI-designated cancer centers; many of the efforts mentioned herein can be observed in academic settings. However, this approach is essential for NCI-designated cancer centers.

## Innovation for the Future of Cancer Science and Technology

The third goal of SRM teams focuses on innovation, ensuring that SRs not only support current research but also drive future scientific advances. This goal involves the development of internal research and development (R&D) within cores and the establishment of dedicated innovation platforms. These efforts are realized through pilot studies, method development, and scaling novel approaches into routine services. This iterative process ensures that emerging technologies are robust, reproducible, and broadly accessible.

Cancer centers have adopted various models to support R&D within SRs. Some cancer centers have dedicated funds (available through internal grant competitions) to support these efforts, while others collaborate with cancer center members or technology providers. Alternative models to support innovation include institutionally supported laboratories that serve as incubators for cutting-edge methodologies. These labs bridge the gap between experimental development and operational implementation, thus enabling the rapid translation of new technologies into SR services.

These activities and innovation laboratories, which are typically managed by SRM teams, must align with CCSG expectations for forward-looking, high-impact research infrastructure. These coordinated efforts are the secret ingredient for a successful “bench-to-bedside and back” transition.

## Centralized Core Management in Academic Institutions and Cancer Centers

Over the past decade, many academic institutions have transitioned from decentralized to centralized models of core facility management due to the benefits of coordinated oversight. Across both universities and cancer centers, centralized management aims to optimize resource utilization, support financial sustainability, enhance service quality, and foster technological innovation. However, within NCI-designated cancer centers, SRM teams assume an additional level of responsibility. Beyond ensuring efficient operations, these teams must demonstrate that each resource provides measurable value to its investigators and is closely aligned with the cancer center’s scientific mission and strategic priorities. During the CCSG renewal process, which is conducted every five years, SRs are evaluated based not only on their operational performance but also on their scientific impact, utilization, and contribution to the cancer center’s research enterprise. Ultimately, for a cancer center to continue receiving CCSG funding and to maintain its NCI designation, SRM teams must provide compelling evidence that these resources are indispensable to the center’s success and its ability to advance cancer research.

## Conclusion

The future of SRM will be shaped not only by scientific advances but also by an increasingly dynamic and uncertain research environment. Emerging technologies (e.g., single-cell multiomics, spatial biology, artificial intelligence, gene editing, personalized cancer vaccines) are creating unprecedented opportunities for discovery and translation. Simultaneously, shifts in federal funding priorities, evolving regulatory frameworks, changes in public policy, and economic pressures may determine which technologies receive long-term support and adoption. Therefore, SRM teams face a fundamental challenge: making strategic investments in infrastructure, instrumentation, and personnel while anticipating an uncertain future.

Unlike individual research projects, SRs represent long-term institutional commitments. Decisions to establish a new platform often require substantial investments in equipment, specialized expertise, and space. Consequently, SRM teams must continuously evaluate emerging technologies, balance innovation with sustainability, and remain sufficiently agile to adapt to changing scientific priorities. Examples include the growing interest in personalized cancer vaccines and mRNA-based therapeutics, advances in computational and artificial intelligence-driven approaches, and ongoing efforts to reduce, refine, and replace animal models through organoids, organ-on-chip systems, and other alternative experimental platforms. Regardless of whether these technologies become dominant or remain complementary approaches, cancer centers must be prepared to support their investigators while maintaining efficient and sustainable operations.

In this environment, the role of SRM teams extends beyond operational oversight. These teams must be characterized by strategic vision, careful risk assessment, and the ability to align technological investments with both investigators’ immediate needs and a cancer center’s long-term mission. Institutions that successfully balance innovation, flexibility, and sustainability will be best positioned to support groundbreaking cancer research and maintain scientific leadership in an increasingly complex landscape.

### Conflict of Interest

The Authors declare no conflicts of interest.
